# Human shields mediate sexual conflict in a top predator

**DOI:** 10.1098/rspb.2016.0906

**Published:** 2016-06-29

**Authors:** S. M. J. G. Steyaert, M. Leclerc, F. Pelletier, J. Kindberg, S. Brunberg, J. E. Swenson, A. Zedrosser

**Affiliations:** 1 Department of Ecology and Natural Resource Management, Norwegian University of Life Sciences, 1432 Ås, Norway; 2 Faculty of Arts and Sciences, Department of Environmental and Health Studies, University College of Southeast Norway, 3800 Bø, Norway; 3 Département de biologie, Université de Sherbrooke, Sherbrooke, Québec, Canada J1K 2R1; 4 Department of Wildlife, Fish and Environmental Studies, Swedish University of Agricultural Sciences, 90183 Umeå, Sweden; 5 Norwegian Institute for Nature Research, 7485 Trondheim, Norway; 6 Institute of Wildlife Biology and Game Management, University of Natural Resources and Life Sciences, 1180 Vienna, Austria

**Keywords:** human shield, fear ecology, resource selection, safety refuge, sexual conflict, sexually selected infanticide

## Abstract

Selecting the right habitat in a risky landscape is crucial for an individual's survival and reproduction. In predator–prey systems, prey often can anticipate the habitat use of their main predator and may use protective associates (i.e. typically an apex predator) as shields against predation. Although never tested, such mechanisms should also evolve in systems in which sexual conflict affects offspring survival. Here, we assessed the relationship between offspring survival and habitat selection, as well as the use of protective associates, in a system in which sexually selected infanticide (SSI), rather than interspecific predation, affects offspring survival. We used the Scandinavian brown bear (*Ursus arctos*) population with SSI in a human-dominated landscape as our model system. Bears, especially adult males, generally avoid humans in our study system. We used resource selection functions to contrast habitat selection of GPS-collared mothers that were successful (i.e. surviving litters, *n* = 19) and unsuccessful (i.e. complete litter loss, *n* = 11) in keeping their young during the mating season (2005–2012). Habitat selection was indeed a predictor of litter survival. Successful mothers were more likely to use humans as protective associates, whereas unsuccessful mothers avoided humans. Our results suggest that principles of predator–prey and fear ecology theory (e.g. non-consumptive and cascading effects) can also be applied to the context of sexual conflict.

## 1. Introduction

Fear ecology theory predicts that individuals adopt spatio-temporal behavioural strategies to minimize predation risk and, therefore, trade food or other resources for safety [1]. Individuals can use spatial (e.g. habitat type, escape possibilities) or temporal (e.g. daylight regimes, seasonality) cues to assess predation risk and alter their habitat selection accordingly [2,3]. Individuals may also anticipate their predators' habitat use and use areas that are perceived as dangerous by their predators as refuges [4]. Apex predators often instill fear in mesopredators, because apex predators can kill mesopredators as competitors, or exploit them as prey. Thus, the mere presence of an apex predator may suppress mesopredator presence spatio-temporally, thereby creating refuges for the mesopredators' main prey. Such commensal relationships are referred to as ‘protective associations’, and are best documented in birds [5], but occur also in mammals [4]. Among mammals, some of the best examples come from systems in which natural top predators lost their apex role to humans and in which human footprint (i.e. landscape variables associated with humans) acts as a shield against predation (i.e. human shields). For example, Berger [6] found that moose (*Alces alces*) shifted their calving grounds to the immediate vicinity of roads in the Yellowstone ecosystem to avoid offspring predation by traffic-averse brown bears (*Ursus arctos*). Atickem *et al.* [7] demonstrated that mountain nyala (*Tragelaphus buxtoni*) used human shields against their main predator, the spotted hyena (*Crocuta crocuta*), in the Bale Mountains National Park in Ethiopia.

Interspecific predation is usually not an important mortality factor in apex predators [8]. Non-parental infanticide, however, is common in some apex predator species and can act as a strong selective pressure [9]. Sexually selected infanticide (SSI) is a male reproductive strategy in which perpetrators kill unrelated dependent offspring to create mating opportunities with the victimized mothers [10]. SSI is common in polygamous species with prolonged maternal care and occurs in seasonal breeders during the mating season [11,12]. Because SSI can be costly for the victimized mothers, several counterstrategies have evolved against it, such as multi-male mating and multiple paternity litters [13], territoriality and group defence [14], and spatio-temporal avoidance of infanticidal males [15]. The mechanism for avoiding infanticide in space and time should be similar to those of predation avoidance; i.e. individuals should use spatial and temporal cues to anticipate their perpetrators' habitat use and thereby minimize infanticide risk [16].

The brown bear is a good example of a top predator that has lost its apex status to humans in most of its current geographical range. Throughout history, brown bears have been hunted or persecuted by humans, often leading to local extinctions [17]. In populations that persisted or have recovered, humans may have had far-reaching effects on bear behaviour [18] and life history [17]. Infanticide is common in brown bears, although its occurrence and adaptive significance may vary across populations [19]. One commonality among populations, however, is that females with cubs-of-the-year (hereafter ‘females with cubs’) alter their habitat selection to avoid infanticide by males [20,21]. Such avoidance strategies can have negative nutritive effects [20,22,23]. Whether or not such strategies pay off in terms of offspring survival remains, however, unknown.

Understanding the relationships between habitat selection, survival and reproductive success is crucial to advancing our knowledge about ecological and evolutionary dynamics [24], as well as for adaptive management and conservation [25]. We are beginning to understand the adaptive significance of habitat selection in the context of predator–prey dynamics [2,26,27], however, it remains poorly understood in a context of sexual selection [28]. Here, we investigate female habitat selection strategies and their efficiency to reduce SSI, using the brown bear as a model species. We hypothesize (H1) that habitat selection of mothers is an important component of offspring survival and that (H2) humans can act as a protective shield for mothers against SSI. We evaluated our hypotheses in a Scandinavian brown bear population with SSI [11], by contrasting habitat selection of successful and unsuccessful mothers in terms of litter survival during the high risk period for SSI, the mating season.

## 2. Material and methods

### (a) Study system

Our study area encompassed approximately 13 000 km^2^ of intensively managed boreal forest in south-central Sweden (approx. 61° N, 15° E). The area is intersected with a dense network of roads (0.7 and 0.14 km km^−2^ forest roads and high traffic roads, respectively). Human population density is among the lowest in the European brown bear range, with humans concentrated in villages (greater than or equal to 200 inhabitants) in the northern and southern parts of the study area. Small settlements (less than 200 permanent residents) and isolated houses are, however, scattered throughout the area [29].

Bear population density is approximately 30 individuals per 1000 km^2^ and the population is hunted annually from 21 August–15 October or until quotas are filled [30]. Family groups are protected from hunting. Human-caused mortality is especially high in close proximity to settlements, villages and highly human-accessible terrain [31]. SSI is common in our study population [32,33]. Annual cub mortality averages 35% and occurs predominantly during the mating season (approx. 85%), which lasts from early May to mid-July (electronic supplementary material, S1—figure S1) [19,32]. Infanticide by unrelated males causes at least 92% of that mortality [34] and SSI explains approximately 14% of the variation in population growth [32]. Mothers use various strategies to reduce infanticide risk, such as aggression, multi-male mating [11], as well as spatio-temporal avoidance of infanticidal males during the mating season [16]. Earlier research in our study area documented that females with cubs avoid adult males and to lesser extent solitary females (with whom the males associate) during the mating season by associating with human footprint, among other strategies. Adult males and solitary females strongly avoid human footprint throughout the year [16]. After the mating season, when there is virtually no risk for SSI, females with cubs shift their habitat selection behaviour towards that of conspecifics and strongly avoid human footprint [16]. Congruent with predictions from fear ecology theory [3], spatio-temporal avoidance of SSI has a negative nutritive effect for females with cubs in our study system. Females with cubs have lower diet quality (i.e. lower protein content, higher fibre content) compared with adult males and especially solitary females, but only during the mating season [23]. After the mating season, females with cubs appear to compensate for this nutritive cost and have a higher diet quality compared with conspecifics [23]. Thus, residing close to human settlements cannot be explained as a foraging strategy by bears to obtain human-derived foods in our study system [35–37].

### (b) Bear monitoring

We captured and equipped female brown bears with Global Positioning System Plus collars (GPS; Vectronic Aerospace GmbH) between 2005 and 2012 by aerial darting with an immobilization drug from a helicopter. For details on capture and handling, refer to Arnemo *et al.* [38]. The GPS collars delivered one position per 30 min. We removed all relocations with dilution of precision values ≥5 to improve spatial accuracy [39], which resulted in an average fix rate of 60.6% and 57.0% for successful and unsuccessful females, respectively. Fix rates between successful and unsuccessful mothers were not statistically different (two sample *t*-test, *t*_20.444_ = −0.571, *p* = 0.575; electronic supplementary material, S1—table S1). We do not capture and replace collars of females with cubs for ethical reasons. Consequently, the collars from females that come out of the den often have relatively low battery levels, which could explain the low fix rates.

We monitored the litter presence and cub survival from a helicopter or the ground at least three times per year during the entire study period (2005–2012). We monitored the litter presence and cub survival continuously from the ground during the 2008–2012 mating seasons by recording cub sign (i.e. direct observation, scats, tracks, cub remains) at GPS cluster sites (i.e. greater than or equal to three consecutive GPS relocations within a 15 m radius of females with cubs). We recorded date of litter loss when we found cub remains at GPS cluster sites or estimated the date of loss based on GPS movement patterns of the females and sign of cub presence at GPS cluster sites [34].

### (c) Spatial landscape data

We derived spatial landscape data known to be important in brown bear behaviour (e.g. [16,29]) from three source layers. For each 25 × 25 m cell in the study area, we used digital topographical maps to derive the Euclidean distance (kilometre) to the nearest human habitation (defined as villages, settlements and isolated houses), forest road and high-traffic road (hereafter ‘road’). In addition, we used the topographical maps to derive non-forested land cover types (bogs and tree-rich bogs, hereafter ‘TRB’). We used Resourcesat-1 (IRSP-LISS3) imagery captured in 2007 to calculate the normalized difference vegetation index (NDVI, a proxy for vegetation density) for each 23.5 × 23.5 m pixel in the study area, following Lillesand *et al.* [40]. We used the 2010 Swedish National Forest Inventory map (50 × 50 m pixels, available at www.slu.se) to derive the land cover types: ‘clearcut’ (forest age <10 years or average tree height: less than 2 m), ‘young forest’ (forest age = 10–29 years or average tree height: 2–7 m), ‘mid-aged forest’ (forest age = 30–75 years or average tree height: 8–15 m) and ‘old forest’ (forest age >75 years or average tree height: greater than 15 m). Refer to the electronic supplementary material, S2 for a theoretical motivation for the consideration of the landscape variables in our models.

### (d) Data analyses

We contrasted the resource selection of successful (16 individuals, 19 bear years) and unsuccessful (10 individuals, 11 bear years) mothers following the resource selection function approach, in which the GPS relocations represent ‘resource use’, and randomly distributed locations represent ‘resource availability’ [41]. One female experienced partial litter loss and was not included in our analyses. We modelled the resource selection of unsuccessful mothers from 1 May until the date of complete litter loss, and randomly assigned an ‘end date’ for each successful mother according to the density distribution of infanticide, infanticide attempts and cub disappearance in our study area (electronic supplementary material, S1—figure S1). We truncated the study period to the last observed day of litter loss, as observed in our study, to balance the monitoring periods of successful and unsuccessful mothers (electronic supplementary material, S1—table S1). We excluded one successful mother from the analysis because we retrieved her GPS relocations only one day before the end of the study period (electronic supplementary material, S1—table S1). For each individual, we sampled resource availability within their annual 100% minimum convex polygon home range using a number of locations equal to each individuals’ relocations. We linked the used and the available locations to all landscape variables in a geographical information system (ESRI ArcGIS v. 10.1).

We used logistic generalized linear mixed effect models with a logit link function to model resource selection, with resource use (1) versus availability (0) as the response variable [41]. We considered all the landscape variables as fixed effects, and ‘bear identity’ and ‘year’ as random factors on the intercept. The land cover types were included as dummy variables [42]. We defined 15 biologically plausible candidate models *a priori* [43], based on specific combinations of non-collinear landscape variables (variance inflation factors, VIFs < 3) (electronic supplementary material, S1—table S2) [44]. Because we hypothesized that resource selection is an important component of cub survival in female brown bears, we introduced the interaction term ‘litter survival’ (0, litter loss, 1, litter survival) on all, or a specific set of, landscape variables in the candidate models (electronic supplementary material, S1—table S2), and expected that the top-ranked model would contain landscape variables interacting with litter survival [45]. We scaled all continuous variables around a mean = 0 and variance = 1 to facilitate comparison. We used second-order, bias-corrected Akaike information criterion values (AIC_c_), and their differences (ΔAIC_c_) and weights (AIC_cw_) to select the most parsimonious model from the candidates [43]. We evaluated the relative importance of potential interactions between landscape variables and survival in the most parsimonious model by systematically including and excluding the interaction term and recalculating the ΔAIC_c_ relative to the full model (ΔAICc_diff_; negative values indicate support for including the interaction term). Because we expected that mothers could increase litter survival by using human footprint as a shield against SSI, we predicted that the interaction between ‘distance to the nearest habitation’ and ‘cub survival’ would be among the most influential model terms. We considered models and model terms with ΔAIC_c_ values ≥4 as inconclusive [43]. We reversed the sign of parameter estimates of the ‘distance to’ variables to facilitate interpretation; positive values then indicated ‘selection for’ whereas negative values indicated ‘avoidance’. We used R v. 3.1.1. software for all statistical analyses [46], and used the package ‘lme4’ to fit the mixed effect models [47].

## 3. Results

We determined the date of complete litter loss by 10 females in 11 cases. We located cub remains in five cases and determined the date of litter loss based on the absence of cub sign at cluster locations of the mothers. In six more cases, we based the date of complete litter loss on the mothers' movement patterns [34]. The dates of litter loss ranged between 8 May and 16 June, and we obtained an average of 546 relocations per unsuccessful mother (range: 84–924). We obtained relocation data from 16 successful mothers during 18 bear years, which were randomly assigned an ‘end date’ ranging between 6 May and 16 June (mean *N*_relocations_ = 831, range: 142–1575). There was no collinearity among the landscape variables.

**Figure 1.**
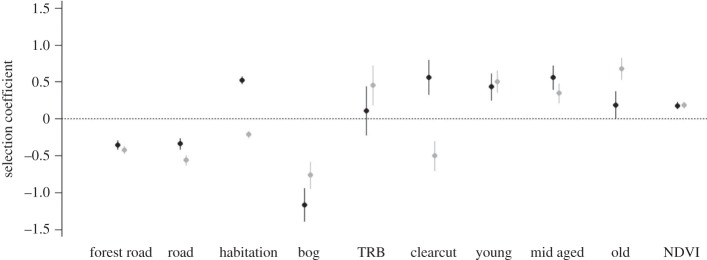
Parameter estimates and confidence intervals (*β* ± 1.96 s.e.) of model variables included in the most parsimonious model to evaluate resource selection of female brown bears that experienced litter survival (black) and complete litter loss (grey) during the mating season in south-central Sweden (2005–2012). We scaled all continuous variables around mean = 0 and variance = 1 to facilitate comparison and reversed the sign of the ‘distance to’ variables to aid interpretation. Positive values indicate selection, negative values indicate avoidance. NDVI, normalized difference vegetation index; TRB, tree-rich bog; old, old forest; mid aged, mid-aged forest; young, young forest.

The full model, including all landscape variables interacting with ‘litter survival’, was the most parsimonious model (AIC_cw_ = 1; electronic supplementary material, S1—table S2). All other candidate models were inconclusive (ΔAIC_c_ ≥ 20.8; electronic supplementary material, S1—table S3). Unsuccessful mothers (i.e. the main effects of the landscape variables in the most parsimonious model) avoided forest roads (*β* ± s.e.: −0.420 ± 0.025), human habitation (−0.210 ± 0.022), roads (−0.557 ± 0.031), bogs (−0.761 ± 0.091) and clearcuts (−0.501 ± 0.102). They selected for tree-rich bogs (0.452 ± 0.135), young forest (0.504 ± 0.075), mid-aged forest (0.348 ± 0.066), old forest (0.675 ± 0.074) and patches with high NDVI values (0.188 ± 0.020) (figure 1; electronic supplementary material, S1—table S3). Successful mothers (i.e. the main effects of the landscape variables in the most parsimonious model + the interaction term ‘survival’) avoided forest roads (*β* ± s.e.: −0.349 ± 0.030), roads (−0.338 ± 0.036) and bogs (−1.159 ± 0.114). Successful mothers selected for human habitation (0.524 ± 0.027), clearcuts (0.560 ± 0.117), young forest (0.437 ± 0.091), mid-aged forest (0.557 ± 0.081), old forest (0.190 ± 0.090) and patches with high NDVI values (0.182 ± 0.024). Tree-rich bogs did not affect habitat selection of successful mothers (0.188 ± 0.165) (figure 1; electronic supplementary material, S1—table S3).

**Figure 2.**
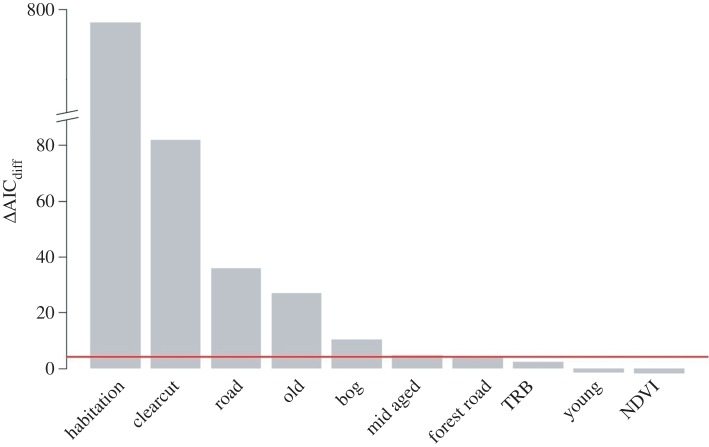
Relative importance (ΔAICc_diff_) of the interaction term ‘litter survival’ on the landscape variables in the most parsimonious model to assess resource selection of female brown bears that experience litter survival and complete loss during the mating season in south-central Sweden (2005–2012). We reversed the sign of the ΔAICc_diff_ values to facilitate interpretation: high values indicate high importance. ΔAICc_diff_ > 4 (horizontal line) supports the inclusion of the interaction term ‘litter survival’ on landscape variables. NDVI, normalized difference vegetation index; TRB, tree-rich bog; old, old forest; mid aged, mid-aged forest; young, young forest. (Online version in colour.)

Habitat selection of successful and unsuccessful females differed (i.e. the interaction term ‘survival’ on the landscape variables in the most parsimonious model) with respect to, and in order of relative importance, distance to habitation (0.734 ± 0.027, ΔAICc_diff_ = −761.7), clearcut (1.061 ± 0.117, ΔAICc_diff_ = −81.9), distance to roads (0.219 ± 0.036, ΔAICc_diff_= −35.8), old forest (−0.485 ± 0.090, ΔAICc_diff_ = −26.9), bogs (−0.398 ± 0.114, ΔAICc_diff_ = −10.2), mid-aged forest (0.210 ± 0.081, ΔAICc_diff_ = −4.7). Habitat selection of successful and unsuccessful females was not different with respect to landscape variables distance to forest roads (0.071 ± 0.030, ΔAICc_diff_ = −3.67), tree-rich bog (−0.341 ± 0.165, ΔAICc_diff_ =−2.27), young forest (−0.067 ± 0.091, ΔAICc_diff_ = 1.47) and NDVI (−0.006 ± 0.024, ΔAICc_diff_ = 1.94) (figures 1 and 2; electronic supplementary material, S1—table S3). Variance components of the random factors ‘bear identity’ and ‘year’ were 0.151 and 0.002, respectively.

Variation in resource availability had little effect on our results. First, resource availability for successful and unsuccessful mothers was similar (electronic supplementary material, S1—tables S4–6). Second, resampling resource availability over the entire study area yielded near identical results (second order habitat selection, electronic supplementary material, S3). Third, irrespective of resource availability, habitat use differed between successful and unsuccessful mothers, with successful mothers having greater exposure to human footprint than unsuccessful mothers (electronic supplementary material, S4).

## 4. Discussion

Our study produced two key findings. First, we showed that habitat selection was an important component of offspring survival in female brown bears (H1). Second, we showed that human footprint could mediate sexual conflict, with humans acting as protective associates for female brown bears against SSI (H2). Variation in resource availability and sampling scale had little effect on our results.

The most influential factor that differentiated habitat selection between successful and unsuccessful mothers was ‘distance to human habitation’; successful mothers strongly selected for areas in relative close proximity to human habitation (median distance = 783 m, median distance random locations = 1070 m), whereas unsuccessful mothers avoided such areas (median distance = 1213 m, median distance random locations = 1035 m) (figures 1 and 2; electronic supplementary material, S1—tables S4–6). Other differences in habitat selection (e.g. with respect to clearcuts, forest types, roads) between successful and unsuccessful mothers were more subtle. Generally, habitat selection of successful and unsuccessful mothers did not match earlier documented patterns in habitat selection of adult male bears during the mating season in our study system [16]. We are confident that the low fix rates did not affect our main results, because there was no difference in fix rates between successful and unsuccessful mothers, and there was no strong relationship between NDVI (vegetation density and canopy cover can hamper GPS fix rates) and proximity to human habitation (electronic supplementary material, S1—figure S2).

Congruent with previous research in our study system [16,23], our results provide strong evidence that female brown bears in Scandinavia avoid adult males to reduce the risk for SSI, and that using human shields can pay off in terms of offspring survival. A similar mechanism was suggested in a Canadian brown bear population, in which humans temporally displace adult males at prime pink salmon (*Oncorhynchus gorbuscha*) fishing spots, and thereby create temporal refuges for females with cubs [48].

An increasing number of studies have shown that habitat selection is an important component of survival in a predator-driven landscape [24]. For example, habitat selection patterns towards open habitat types or human infrastructures influences survival or reproductive success in several ungulates [26,27,49]. Our results clearly show that habitat selection is an important component of survival also in an apex predator, the brown bear, but is driven by sexual selection and conflict rather than predator–prey dynamics. This implies that predictions from fear ecology theory, which are typically framed in a context of predator–prey dynamics, can be expanded to sexual selection theory and sexual conflict. Infanticide is common among species that express strong sexual selection [12,13]. In such species, infanticidal males can be considered to play the ecological role of a predator and mothers with dependent offspring the role of prey. Individuals vulnerable to infanticide benefit from hiding behind the shield of a local apex predator. In analogy with predator–prey theory, infanticide should thus invoke direct numerical effects [32], as well as risk effects (e.g. food or safety trade-offs, physiological stress) [15,23] and density—and behaviour-mediated effects on third parties (e.g. cascading effects and trophic interactions) [2].

The use of a protective associate is typically considered as a commensal relationship, implying that one party (the ‘protected’) mostly benefits from the association, whereas the other party experiences no significant costs or benefits [5]. Using a protective associate, however, can also be costly. Protective associates can be dangerous and may kill individuals that seek protection, or disturb them and incur physiological stress [5]. Also, density-dependent mechanisms, such as increased resource competition and elevated parasite burdens, may occur if the protected individuals or species aggregate in relatively high densities around a protective associate [50]. Our study illustrates that human footprint can influence behavioural strategies that affect survival and reproductive success. However, whether or not such behaviour is adaptive remains unclear, because it is unknown how potential costs (e.g. elevates stress, mortality risk) of associating with humans relates to its benefits.

The current wildlife management paradigm postulates that bears sometimes associate with humans to obtain good quality and easily accessible foods (rubbish, compost, etc.) (reviewed in [37]). However, there is increasing evidence that males are important drivers of the socio-spatial organization of bear populations, and not only foraging strategies [35–37]. Several studies have shown that females with cubs trade food for safety by spatio-temporally avoiding adult males [22,23]. Our results concur with these findings, and we suggest that mothers and other vulnerable bears seek safety near humans primarily to avoid potentially aggressive males, rather than being attracted to humans because of good foraging opportunities.

## 5. Conclusion

Our results showed that habitat selection is an important component of offspring survival in a species in which sexual conflict is the foremost important cause of offspring mortality. This means that predictions from fear ecology theory can be extended from a purely predator–prey framework to sexual selection theory in species exhibiting strong sexual conflict. We also provide evidence that female bears can increase their reproductive success by using human footprint as a shield against infanticide. Using protective associates is an intriguing, but poorly understood aspect of behavioural ecology, especially regarding how potential costs (e.g. stress) of associating can affect individual performance, as well as the ecological and evolutionary consequences of such associations.

## Supplementary Material

Electronic Supplementary Material 1 - Supporting tables and figures

## Supplementary Material

Electronic Supplementary Material 2: Spatial landscape data - motivation and predictions

## Supplementary Material

Electronic Supplementary Material 3 - Second-order habitat selection

## Supplementary Material

Electronic Supplementary Material 4 - Habitat use

## Data accessibility

The data for this manuscript are available at Dryad: http://dx.doi.org/10.5061/dryad.p5n87.
