# Supplementary material for: Human shields mediate sexual conflict in a top predator
Source: Proc Biol Sci. 2016 Jun 29;283(1833):20160906. doi: 10.1098/rspb.2016.0906 (PMC4936045; doi:10.1098/rspb.2016.0906)
Supplement: Electronic Supplementary Material 1 - Supporting tables and figures [file rspb20160906supp1.pdf]

**Electronic Supplementary Material 1 – Supporting tables and figures**

ESM1 Table S1. Study-unit specific monitoring data of female brown bears with dependent offspring that we monitored in south-central Sweden between 2005 and 2012 to assess effects of habitat selection on litter fate (complete mortality/complete survival of the cubs in the litter). We excluded one female that experienced partial litter loss from the analyses. The start day of the study was 1 May (i.e., start of the mating season). 'End day' indicates the day of complete litter loss, or a randomly assigned date that followed the previously documented distribution of sexually selected infanticide events or attempts on the study area [1]. 'Truncated' implies whether or not we truncated the data of a specific individual to the last day of litter loss (16 June) observed in this study. $N_{relocations}$ and GPS relocation fix rates were computed after conservative GPS data screening to retain only highly accurate GPS relocations. Fix rates between females that experienced litter survival (60.6%) and mortality (57.0%) were not statistically different (two Sample t-test, $t = -0.571$, $df = 20.444$, $p = 0.575$). We removed one successful mother from the analysis (*), because we started receiving GPS relocation data one day prior to the end of the truncated study period.

| Bear ID | year | Start day | End day | Truncated | Nlocs | Fix rate | Fate |
|---|---|---|---|---|---|---|---|
| B7 | 2005 | 15 May | 24 May | no | 142 | 0.336 | survival |
| B1 | 2007 | 1 May | 28 May | no | 554 | 0.437 | survival |
| B10 | 2007 | 1 May | 8 June | no | 980 | 0.549 | survival |
| B4 | 2008 | 6 May | 5 June | no | 765 | 0.531 | mortality |
| B5 | 2008 | 6 May | 5 June | no | 553 | 0.384 | mortality |
| B19 | 2008 | 6 May | 8 May | no | 84 | 0.875 | mortality |
| B22 | 2008 | 6 May | 27 May | no | 619 | 0.627 | survival |
| B2 | 2009 | 1 May | 15 June | no | 1499 | 0.694 | mortality |
| B4 | 2009 | 1 May | 8 May | no | 201 | 0.598 | mortality |
| B11 | 2009 | 1 May | 15 June | no | 500 | 0.744 | mortality |
| B8 | 2009 | 1 May | 6 June | no | 890 | 0.526 | survival |
| B17 | 2009 | 1 May | 30 June | yes | 1916 | 0.679 | survival |
| B20 | 2009 | 1 May | 14 June | no | 1256 | 0.607 | survival |
| B21 | 2009 | 1 May | 24 May | no | 906 | 0.838 | survival |
| B13 | 2010 | 1 May | 3 June | no | 924 | 0.583 | mortality |
| B16 | 2010 | 1 May | 16 May | no | 468 | 0.650 | mortality |
| B9 | 2010 | 1 May | 11 May | no | 410 | 0.872 | survival |
| B14 | 2010 | 1 May | 5 June | no | 1156 | 0.703 | survival |
| B23 * | 2010 | 14 June | 19 June | yes | 134 | 0.570 | Survival |
| B22 | 2011 | 1 May | 6 June | no | 632 | 0.366 | mortality |
| B3 | 2011 | 1 May | 19 June | yes | 868 | 0.377 | survival |
| B7 | 2011 | 1 May | 24 June | yes | 1113 | 0.439 | survival |
| B16 | 2011 | 1 May | 6 June | no | 809 | 0.478 | survival |
| B19 | 2011 | 1 May | 10 May | no | 330 | 0.780 | survival |
| B18 | 2012 | 1 May | 29 May | no | 405 | 0.301 | mortality |
| B20 | 2012 | 1 May | 30 May | no | 757 | 0.544 | mortality |
| B6 | 2012 | 1 May | 22 June | yes | 1768 | 0.723 | survival |
| B9 | 2012 | 1 May | 21 May | no | 837 | 0.890 | survival |
| B12 | 2012 | 1 May | 14 June | no | 933 | 0.451 | survival |
| B15 | 2012 | 21 May | 4 June | no | 426 | 0.647 | survival |

ESM1 Table S2. Candidate models for third-order resource selection functions of female brown bears during the mating season in south-central Sweden (2005-2012), ranked according to the second-order, bias-corrected Akaike Information Criterion difference ($\Delta AIC_c$) and weight ($AIC_{cw}$) values. '✓' indicates the inclusion of a landscape variable as a main term in a candidate model, '*' indicates the inclusion of the interaction term 'litter survival' with a certain landscape variable. NDVI = Normalized Difference Vegetation Index.

| Model selection diagnostics | | | Landscape variables | | | | | | | | | |
|---|---|---|---|---|---|---|---|---|---|---|---|---|
| Rank | ΔAICc | AICcw | Distance to the nearest *forest road* | *road* | *habitation* | NDVI | Bog | Tree-rich bog | Clearcut | Young forest | Mid-aged forest | Old forest |
| 1 | - | 1 | ✓ * | ✓ * | ✓ * | ✓ * | ✓ * | ✓ * | ✓ * | ✓ * | ✓ * | ✓ * |
| 2 | 20.8 | 0 | ✓ | ✓ * | ✓ * | ✓ * | ✓ | ✓ | ✓ * | ✓ * | ✓ * | ✓ * |
| 3 | 53.0 | 0 | ✓ * | ✓ * | ✓ * | ✓ * | ✓ | ✓ | ✓ * | ✓ | ✓ | ✓ * |
| 4 | 202.7 | 0 | ✓ | ✓ | ✓ * | ✓ | ✓ | ✓ | ✓ | ✓ | ✓ | ✓ * |
| 5 | 1206.1 | 0 | ✓ | ✓ | ✓ | ✓ | ✓ | ✓ | ✓ | ✓ | ✓ | ✓ |
| 6 | 2028.9 | 0 | ✓ * | ✓ * | ✓ * | | | | | | | |
| 7 | 2036.7 | 0 | ✓ | ✓ * | ✓ * | | | | | | | |
| 8 | 2065.0 | 0 | ✓ | ✓ | ✓ * | | | | | | | |
| 9 | 2477.2 | 0 | | | | ✓ | ✓ * | ✓ * | ✓ * | ✓ * | ✓ * | ✓ * |
| 10 | 2479.1 | 0 | | | | ✓ * | ✓ * | ✓ * | ✓ * | ✓ * | ✓ * | ✓ * |
| 11 | 2526.5 | 0 | | | | ✓ * | ✓ | ✓ | ✓ * | ✓ * | ✓ * | ✓ * |
| 12 | 2562.6 | 0 | | | | ✓ * | ✓ | ✓ | ✓ * | ✓ | ✓ | ✓ * |
| 13 | 2848.1 | 0 | | | | ✓ | ✓ | ✓ | ✓ | ✓ | ✓ | ✓ |
| 14 | 2921.4 | 0 | ✓ | ✓ | ✓ | | | | | | | |
| 15 | 4478.4 | 0 | | | | | | | | | | |

ESM1 Table S3. Most parsimonious candidate model to evaluate third-order resource selection of female brown bears in relation to litter fate (survival/mortality) during the mating season in south-central Sweden (2005-2012). NDVI = Normalized Difference Vegetation Index. ‘*’ indicates statistically significant model terms (i.e., 0 not included in the 95% confidence interval). Note that we reversed the sign of parameter estimates of the ‘distance to’ variables to facilitate interpretation; positive values indicated ‘selection for’ whereas negative values indicated ‘avoidance’.

| **Model term** | **β** | **σ** | **95% confidence interval** | | |
|---|---|---|---|---|---|
| | | | ***Lower level*** | ***Upper level*** | |
| Intercept | -0.323 | 0.109 | -0.537 | -0.109 | * |
| Survival vs. Mortality | 0.023 | 0.087 | -0.148 | 0.194 | |
| Distance to the nearest forest road | -0.420 | 0.025 | 0.370 | 0.470 | * |
| Distance to the nearest road | -0.557 | 0.031 | 0.496 | 0.618 | * |
| Distance to the nearest habitation | -0.210 | 0.022 | 0.167 | 0.253 | * |
| Bog (1 vs 0) | -0.761 | 0.091 | -0.939 | -0.583 | * |
| Tree-rich bog (1 vs 0) | 0.452 | 0.135 | 0.187 | 0.718 | * |
| Clearcut (1 vs 0) | -0.501 | 0.102 | -0.700 | -0.302 | * |
| Young forest (1 vs 0) | 0.504 | 0.075 | 0.357 | 0.650 | * |
| Mid-aged forest (1 vs 0) | 0.348 | 0.066 | 0.217 | 0.478 | * |
| Old forest (1 vs 0) | 0.675 | 0.074 | 0.530 | 0.819 | * |
| NDVI | 0.188 | 0.020 | 0.150 | 0.227 | * |
| Distance to the nearest forest road * Survival | 0.071 | 0.030 | -0.130 | -0.013 | * |
| Distance to the nearest road * Survival | 0.219 | 0.036 | -0.290 | -0.149 | * |
| Distance to the nearest habitation * Survival | 0.734 | 0.027 | -0.787 | -0.681 | * |
| Bog * Survival | -0.398 | 0.114 | -0.621 | -0.174 | * |
| Tree-rich bog * Survival | -0.341 | 0.165 | -0.665 | -0.017 | * |
| Clearcut * Survival | 1.061 | 0.117 | 0.831 | 1.291 | * |
| Young forest * Survival | -0.067 | 0.091 | -0.245 | 0.111 | |
| Mid-aged forest * Survival | 0.210 | 0.081 | 0.051 | 0.369 | * |
| Old forest * Survival | -0.485 | 0.090 | -0.662 | -0.307 | * |
| NDVI * Survival | -0.006 | 0.024 | -0.053 | 0.040 | |

ESM1 Table S4: Descriptive statistics of continuous model terms included in the most parsimonious model to assess habitat selection of successful (i.e., experiencing complete litter survival) and unsuccessful (i.e., experiencing loss of the entire litter) female brown bears during the mating season in south-central Sweden (2005-2012). Note that NVDI (Normalized Difference Vegetation Index) is a unitless index, whereas the other covariates are expressed in kilometers. Use and availability were sampled in a 1:1 ratio of GPS relocations and random locations, respectively, and within annual individual-based 100% MCP home ranges.

**Successful females**

| ***Covariate*** | | ***Mean*** | ***Median*** | ***Minimum*** | ***Maximum*** |
|---|---|---|---|---|---|
| Distance to the nearest forest road | *use* | 0.660 | 0.525 | 0.000 | 3.060 |
| | *availability* | 0.605 | 0.456 | 0.000 | 4.364 |
| Distance to the nearest road | *use* | 5.026 | 3.932 | 0.000 | 12.951 |
| | *availability* | 4.581 | 3.966 | 0.000 | 14.812 |
| Distance to the nearest habitation | *use* | 0.979 | 0.783 | 0.025 | 3.083 |
| | *availability* | 1.179 | 1.070 | 0.000 | 3.784 |
| NDVI | *use* | 0.422 | 0.425 | -1.000 | 1.000 |
| | *availability* | 0.392 | 0.402 | -1.000 | 1.000 |

**Unsuccessful females**

| ***Covariate*** | | ***Mean*** | ***Median*** | ***Minimum*** | ***Maximum*** |
|---|---|---|---|---|---|
| Distance to the nearest forest road | *use* | 0.624 | 0.467 | 0.000 | 2.260 |
| | *availability* | 0.487 | 0.460 | 0.000 | 2.525 |
| Distance to the nearest road | *use* | 5.880 | 5.274 | 0.000 | 15.080 |
| | *availability* | 5.127 | 4.572 | 0.000 | 16.086 |
| Distance to the nearest habitation | *use* | 1.128 | 1.213 | 0.025 | 3.116 |
| | *availability* | 1.090 | 1.035 | 0.000 | 3.559 |
| NDVI | *use* | 0.431 | 0.430 | -1.000 | 1.000 |
| | *availability* | 0.395 | 0.405 | -1.000 | 1.000 |

ESM1 Table S5: Proportion of use and availability of landscape variables included as dummies in the most parsimonious model to assess habitat selection of successful (i.e., experiencing complete litter survival) and unsuccessful (i.e., experiencing loss of entire litter) female brown bears during the mating season in south-central Sweden (2005-2012). Use and availability were sampled in a 1:1 ratio of GPS relocations and random locations, respectively, and within annual individual-based 100% MCP home ranges.

| **Land cover type** | **Successful mothers** | | **Unsuccessful mothers** | |
|---|---|---|---|---|
| | ***Use (%)*** | ***Availability (%)*** | ***Use (%)*** | ***Availability (%)*** |
| Bog | 2.6 | 12.0 | 4.6 | 10.1 |
| Tree-rich bog | 1.6 | 3.1 | 2.1 | 2.4 |
| Clearcut | 9.8 | 8.3 | 3.4 | 8.9 |
| Young forest | 17.5 | 16.5 | 18.3 | 16.0 |
| Mid-aged forest | 48.0 | 37.4 | 43.6 | 40.5 |
| Old forest | 14.6 | 16.5 | 23.0 | 15.2 |

ESM1 Table S6: Comparison of resource availability within home ranges of successful (i.e., experiencing complete litter survival) and unsuccessful (i.e., experiencing loss of entire litter) female brown bears during the mating season in south-central Sweden (2005-2012). We tested if resource availability differed between successful and unsuccessful mother with mixed effect regression models with the landscape covariate as response variable. We fitted linear models for the continuous landscape covariates and logistic models for the binomial land cover classes as response variables and 'survival' (1 vs 0) as the only fixed effect. We included 'bear identity' and 'year' as random factors on the intercept. '*' indicates statistically significant model terms (i.e., 0 not included in the 95% confidence interval).

| **Landscape covariate** | **β** | **σ** | **95% confidence interval** | | |
|---|---|---|---|---|---|
| | | | ***Lower level*** | ***Upper level*** | |
| Distance to the nearest forest road | -0.031 | 0.043 | -0.115 | 0.053 | |
| Distance to the nearest road | -0.322 | 0.041 | -0.402 | -0.242 | * |
| Distance to the nearest habitation | -0.085 | 0.051 | -0.185 | 0.015 | |
| Bog (1 vs 0) | -0.173 | 0.088 | -0.345 | -0.001 | * |
| Tree-rich bog (1 vs 0) | 0.089 | 0.157 | -0.219 | 0.397 | |
| Clearcut (1 vs 0) | 0.266 | 0.096 | 0.078 | 0.454 | * |
| Young forest (1 vs 0) | -0.146 | 0.083 | -0.309 | 0.017 | |
| Mid-aged forest (1 vs 0) | 0.037 | 0.064 | -0.089 | 0.162 | |
| Old forest (1 vs 0) | 0.026 | 0.075 | -0.121 | 0.173 | |
| NDVI | 0.004 | 0.032 | -0.059 | 0.067 | |

ESM1 Figure S1. Length and intensity of the mating season (gray) of brown bears in south-central Sweden, based on the density distribution of interactions (i.e., co-occurence within 30 m) between GPS-collared adult males and females. We recorded 96 unique male-female pairs, yielding 6,475 interactions between 2007 and 2011. The density distribution of sexually selected infanticide (N = 20), infanticide attempts (N = 2), and cub loss due to reasons unknown (N = 2) (red) matches well with the mating season of the study population. Figure reproduced from Steyaert [1].

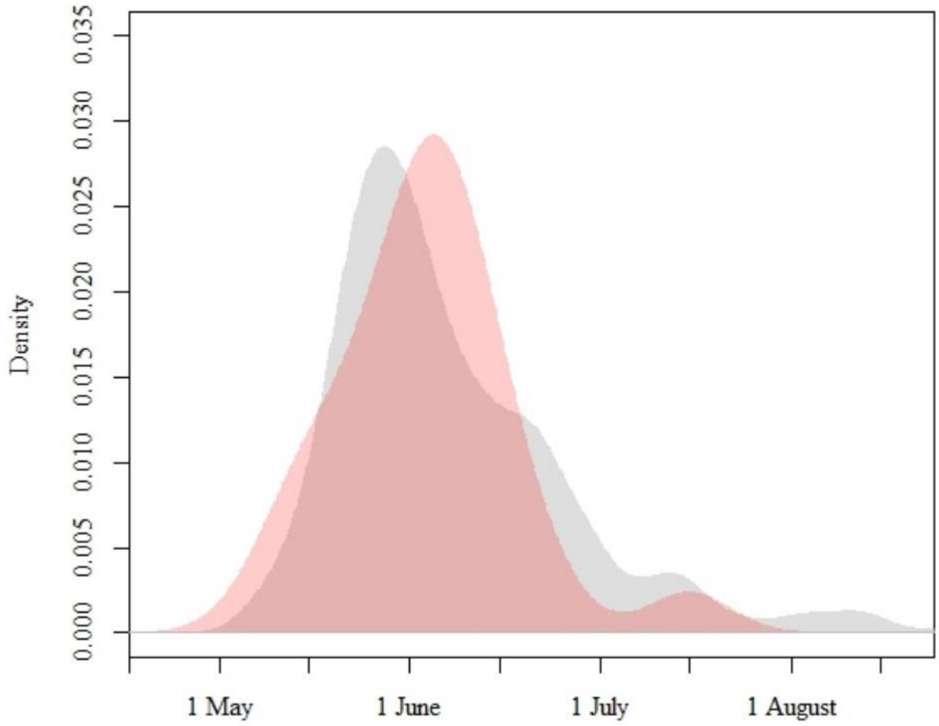

ESM1 Figure S2. Plotting normalized difference vegetation index (NDVI) values (as a proxy for vegetation density which may ) against distance to the nearest habitation (km) of all random locations used in this study revealed no strong relationship between the two covariates (Pearson correlation coefficient r = -0.07).

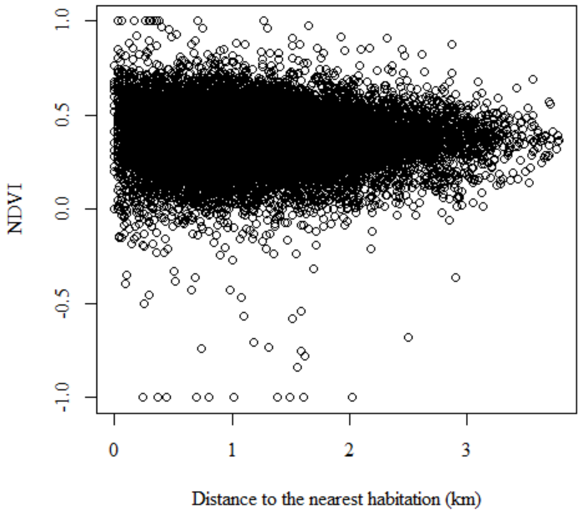


## References

[1] Steyaert, S.M.J.G. 2012 *The mating system of the brown bear in relation to the sexually selected infanticide theory*. Aas, Norway, PhD thesis, Norwegian University of Life Sciences.
