# Supplementary material for: Human shields mediate sexual conflict in a top predator
Source: Proc Biol Sci. 2016 Jun 29;283(1833):20160906. doi: 10.1098/rspb.2016.0906 (PMC4936045; doi:10.1098/rspb.2016.0906)
Supplement: Electronic Supplementary Material 2: Spatial landscape data - motivation and predictions [file rspb20160906supp2.pdf]

Electronic Supplementary Material 2: Spatial landscape data – motivation and predictions

We considered three groups of spatial landscape variables in our analyses; i.e., land cover data, distance to human footprint variables, and the Normalized Difference Vegetation Index. We chose not to include other potentially relevant covariates for animal habitat selection in the candidate models (e.g. topography, distance to habitat edges, patch sizes, etc.), because our main focus was on the land cover data and the human footprint-related variables and because we wanted to avoid overfitting the models [1].

Land cover

Bog and tree-rich bog – Bogs and tree-rich bogs are among the least productive habitat types in the boreal forest in terms of living conditions or foods for many species, including large mammals [2, 3]. Several studies have suggested that female brown bears with dependent offspring avoid high-quality habitat in terms of foraging and energy gain (e.g., prime salmon spawing streams) [4-7], and that trait-mediated effects of infanticide can have a nutritive cost [8, 9]. Because bogs and tree-rich bogs have little to offer in terms of food for solitary adult females and the adult males that associate with them during the mating season [10, 11], we expected that bogs and tree-rich bogs would provide relatively safe habitat for mothers with a dependent litter. Consequently, we expected that successful mothers (i.e. no litter loss during the mating season) would have selected for bogs and tree rich-bogs and that unsuccessful females would have avoided these land cover types.

*Clearcut*s –We expected that successful mothers would have shown strong positive selection for clearcuts, whereas we expected strong avoidance of clearcuts by unsuccessful mothers. Three complementary mechanism form the basis for this prediction. First, besides carpenter ants

(*Camponotus herculeanus)* [12] and some early herbs, grasses, and sedges, recent clearcuts (< 10 years old) have little to offer in terms of food resources for bears during the mating season. Second, previous research in our study area indicated that solitary females and adult males select for densely vegetated habitat patches that provide much cover, presumably to avoid being detected by humans [13, 14]. Females with dependent offspring, however, appeared to select for less dense vegetation, presumably to avoid infanticidal males [11]. Third, recent clearcuts may reflect a stronger human footprint than older forests types, bogs, and tree-rich bogs [15], and adult males and solitary adult females seem to be most sensitive towards human footprint-related landscape features [11, 16, 17]. In summary, we expect that clearcuts would be strongly avoided by solitary females and the males that associate with them during the mating season, because clearcuts are poor habitat in terms of food resources and cover, and have a strong human footprint. Consequently, clearcuts can provide relatively safe habitat for successful mothers, and mothers that had avoided clearcuts were expected to have higher odds of losing their litter due to SSI.

Young forest – Young forest types are typically dense and provide much cover. Previous research in our study area showed that young forest is a highly selected resource for solitary females and adult males during daytime throughout the year, presumably to minimize the risk of disturbance by humans [11, 13, 14, 18]. Because we expect that avoiding conspecifics to reduce the risk for SSI can pay off in terms of offspring survival, we expected that successful mothers would have more strongly avoided young forest than unsuccessful mothers.

Mid-aged and old forest – Brown bear cubs often climb trees when sudden risk appears, for example when approached by humans or conspecifics [11, 19]. The availability of large trees may thus be a critical resource to facilitate escape from SSI for mothers and their litters. Such

trees are more common in more mature forest stands. Therefore, we expected that successful mothers would have more strongly selected for mid-aged and especially old forest types compared to unsuccessful mothers.

Human footprints

As outlined in the main body of our article, we expect that successful mothers associate with humans to reduce the risk for SSI. We considered roads, forest roads, and human habitation (buildings, settlements, villages) as landscape features with a strong human footprint. We distinguished between these three landscape features, because of their varying predictability of human activity, since predictability of a risk or disturbance can be an important cue for animals to adjust their behavior and space use [20-22]. We assumed that human activity is relatively constant and predictable around habitation, less constant but relatively predictable around paved roads with regular motorized traffic, and relatively low and unpredictable on unpaved forest roads. Consequently, we expected that successful mothers would have selected for areas close to, in order of relevance, human habitation, roads, and forest roads. We expected that unsuccessful mothers would generally have avoided these land cover types.

NDVI

The spectral normalized difference vegetation index (NDVI) is a proxy for vegetation density and thus cover [23]. Several wildlife ecological studies have now shown the relevance of NDVI for animal behavior [24, 25], including brown bears [26, 27]. Our previous research indicated that solitary females and adult males strongly select for patches with high NDVI values, especially during daytime [11]. Selection for patches with high NDVI values by successful mothers was stable throughout the day, and lower compared to conspecifics. We previously

explained this behavioral difference as a spatiotemporal anti-SSI strategy. Here, we expected that unsuccessful mothers had selected for patches with higher NDVI values than successful mothers.

References

[1] Zuur, A.F., Ieno, E.N., Walker, N.J., Saveliev, A.A. & Smith, G.M. 2009 *Mixed effects models and extensions in ecology with R*. New York, Springer; 573 p.

[2] Bjørneraas, K., Solberg, E.J., Herfindal, I., Moorter, B.V., Rolandsen, C.M., Tremblay, J.-P., Skarpe, C., Sæther, B.-E., Eriksen, R. & Astrup, R. 2011 Moose Alces alces habitat use at multiple temporal scales in a human-altered landscape. *Wildlife Biology* **17**, 44-54. (doi:10.2981/10-073).

[3] Moen, A. 1999 *National Atlas of Norway: Vegetation*. Hønefoss, Norway200 p.

[4] Rode, K.D., Farley, S.D. & Robbins, C.T. 2006 Sexual dimorphism, reproductive strategy and human activities determine resource use by brown bears. *Ecology* **87**, 2636-2646. (doi:doi:10.1890/0012-9658(2006)87[2636:SDRSAH]2.0.CO;2).

[5] Craighead, J.J., Sumner, J.S. & Mitchell, J.A. 1995 *The grizzly bears of Yellowstone: their ecology in the Yellowstone Ecosystem, 1959 - 1992*. Washington D.C., Island Press.

[6] Pearson, M.A. 1975 *The northern interior grizzly bear Ursus arctos L.* Ottawa, Canada, Canadian Wildlife Service.

[7] Swenson, J.E., Dahle, B. & Sandegren, F. 2001 Intraspecific predation in Scandinavian brown bears older than cubs-of-the-year. *Ursus* **12**, 81-91.

[8] Steyaert, S.M.J.G., Reusch, C., Brunberg, S., Swenson, J.E., Hackländer, K. & Zedrosser, A. 2013 Infanticide as a male reproductive strategy has a nutritive risk effect in brown bears. *Biology Letters*, 20130624. (doi:doi:10.5061/dryad.h359g).

[9] Ben-David, M., Titus, K. & Beier, L. 2004 Consumption of salmon by Alaskan brown bears: a trade-off between nutritional requirements and the risk of infanticide? *Oecologia* **138**, 465-474. (doi:10.1007/s00442-003-1442-x).

[10] Steyaert, S.M.J.G., Endrestøl, A., Hackländer, K., Swenson, J.E. & Zedrosser, A. 2012 The mating system of the brown bear *Ursus arctos*. *Mammal Review* **42**, 12-34. (doi:10.1111/j.1365-2907.2011.00184.x).

[11] Steyaert, S.M.J.G., Kindberg, J., Swenson, J.E. & Zedrosser, A. 2013 Male reproductive strategy explains spatiotemporal segregation in brown bears. *Journal of Animal Ecology* **82**, 836-845. (doi:10.1111/1365-2656.12055).

[12] Frank, S.C., Steyaert, S.M.J.G., Swenson, J.E., Storch, I., Kindberg, J., Barck, H. & Zedrosser, A. 2015 A "clearcut" case? Brown bear selection of coarse woody debris and carpenter ants on clearcuts. *Forest Ecology and Management* **348**, 164-173. (doi:http://dx.doi.org/10.1016/j.foreco.2015.03.051).

[13] Martin, J., Basille, M., Van Moorter, B., Kindberg, J., Allainé, D. & Swenson, J.E. 2010 Coping with human disturbance: spatial and temporal tactics of the brown bear (*Ursus arctos*). *Canadian Journal of Zoology* **88**, 875-883. (doi:doi:10.1139/Z10-053).

[14] Ordiz, A., Støen, O.-G., Delibes, M. & Swenson, J. 2011 Predators or prey? Spatio-temporal discrimination of human-derived risk by brown bears. *Oecologia* **166**, 59-67. (doi:10.1007/s00442-011-1920-5).

[15] Leclerc, M., Dussault, C. & St-Laurent, M.-H. 2014 Behavioural strategies towards human disturbances explain individual performance in woodland caribou. *Oecologia* **176**, 297-306. (doi:10.1007/s00442-014-3012-9).

[16] Nellemann, C., Støen, O.-G., Kindberg, J., Swenson, J.E., Vistnes, I., Ericsson, G., Katajisto, J., Kaltenborn, B.P., Martin, J. & Ordiz, A. 2007 Terrain use by an expanding brown bear population in relation to age, recreational resorts and human settlements. *Biological Conservation* **138**, 157-165. (doi:DOI: 10.1016/j.biocon.2007.04.011).

[17] Elfström, M. & Swenson, J.E. 2009 Effects of sex and age on den site use by Scandinavian brown bears. *Ursus* **20**, 85-93. (doi:10.2192/09gr005.1).

[18] Moen, G.K., Støen, O.-G., Sahlén, V. & Swenson, J.E. 2012 Behaviour of solitary adult Scandinavian brown bears (*Ursus arctos*) when approached by humans on Foot. *PLoS ONE* **7**, e31699. (doi:10.1371/journal.pone.0031699).

[19] Swenson, J.E. 2003 Implications of sexually selected infanticide for the hunting of large carnivores. In *Animal behavior and wildlife conservation* (eds. M. Festa-Bianchet & M. Apollonio), pp. 171-189. Washington DC, Island press.

[20] Lima, S.L. & Bednekoff, P.A. 1998 Temporal variation in danger drives antipredator behavior: The predation risk allocation hypothesis. *The American Naturalist* **153**, 649-659.

[21] Houston, A.I., Higginson, A.D. & McNamara, J.M. 2011 Optimal foraging for multiple nutrients in an unpredictable environment. *Ecology Letters* **14**, 1101-1107. (doi:10.1111/j.1461-0248.2011.01678.x).

[22] Houston, A.I., McNamara, J.M. & Hutchinson, J.M.C. 1993 General results concerning the trade-off between gaining energy and avoiding predation. *Philosophical Transactions of the Royal Society of London. Series B: Biological Sciences* **341**, 375-397. (doi:10.1098/rstb.1993.0123).

[23] Pettorelli, N., Vik, J.O., Mysterud, A., Gaillard, J.M., Tucker, C.J. & Stenseth, N.C. 2005 Using the satellite-derived NDVI to assess ecological responses to environmental change. *Trends in Ecology and Evolution* **20**, 503-510.

[24] Lone, K. 2015 Lidar, habitat structure and the ecology of ungulates in a landscape of fear. Ås, Norway, Norwegian University of Life Sciences.

[25] Rivrud, I.M., Bischof, R., Meisingset, E.L., Zimmermann, B., Loe, L.E. & Mysterud, A. 2016 Leave before it's too late: Anthropogenic and environmental triggers of autumn migration in a hunted ungulate population. *Ecology*, n/a-n/a. (doi:10.1890/15-1191).

[26] Steyaert, S.M.J.G., Støen, O.-G., Elfström, M., Karlsson, J., Lammeren, R.V., Bokdam, J., Zedrosser, A., Brunberg, S. & Swenson, J.E. 2012 Resource selection by sympatric free-ranging dairy cattle and brown bears *Ursus arctos*. *Wildlife Biology* **17**, 389-403. (doi:10.2981/11-004).

[27] Bojarska, K. & Selva, N. 2012 Spatial patterns in brown bear Ursus arctos diet: the role of geographical and environmental factors. *Mammal Review* **42**, 120-143. (doi:10.1111/j.1365-2907.2011.00192.x).
