# Supplementary material for: Human shields mediate sexual conflict in a top predator
Source: Proc Biol Sci. 2016 Jun 29;283(1833):20160906. doi: 10.1098/rspb.2016.0906 (PMC4936045; doi:10.1098/rspb.2016.0906)
Supplement: Electronic Supplementary Material 3 - Second-order habitat selection [file rspb20160906supp3.pdf]

Electronic Supplementary Material 3 – Second-order habitat selection

Habitat selection has a hierarchical nature [1, 2], from the geographical range of a species at the largest scale (first-order selection), spatial and temporal dynamics of home ranges (third-order selection), habitat selection within the home range (second-order selection), to micro-habitat selection at the smallest spatial scale (e.g., selection of food items at a foraging site, fourth-order selection) [1]. Our main focus was third-order habitat selection; i.e. to investigate how mothers select resources once the home ranges have established, and how that selection affected litter fate. However, our analyses did not provide insight in second-order habitat selection; or how the location of the home range within the landscape influenced litter survival. Consequently, and complimentary to the main article, we evaluated the relationship between second-order habitat selection and litter survival in female brown bears. We hypothesized ESM3 H1) that second-order habitat selection is an important determining factor for litter fate, and ESM3 H2) that successful mothers (i.e. experiencing litter survival during the mating season) benefit from selecting for areas near human footprint, whereas unsuccessful mothers avoid such areas.

Methods – We used the same modeling approach as outlines in the main document, with the exception that we now sampled resource availability over the entire study area; i.e. a spatial merge of the 100% minimum convex polygon home ranges of all mothers included in this study.

Results – The full model, including the interaction ‘litter survival’ on all landscape covariates was the most parsimonious model ($\Delta AIC_c = 0$, $AIC_{cw} = 1$) to assess the relationship between second-order habitat selection and litter survival. All other candidate models were inconclusive (all ΔAIC_c values ≥ 56.3) (ESM3 Table 1).

Unsuccessful mothers (i.e., the main effects of the landscape variables in the most parsimonious model) avoided human habitation (-0.116 ± 0.020), roads (-0.375 ± 0.011), bogs (-0.652 ± 0.086), and clearcuts (-0.478 ± 0.101). They selected for tree rich bogs (0.338 ± 0.124), young forest (0.453 ± 0.072), mid-aged forest (0.362 ± 0.065), old forest (0.638 ± 0.072), and patches with high NDVI values (0.184 ± 0.019). Habitat selection of unsuccessful mothers was not affected by forest roads (0.009 ± 0.021) (ESM3 Table 2, ESM3 Figure S1).

The interaction term 'survival' was influential on, in order of relative importance, distance to the nearest human habitation (0.540 ± 0.025, ΔAIC_{diff} = -485.4) and forest road (-0.308 ± 0.025, ΔAIC_{diff} = -146.7), clearcuts (1.144 ± 0.116, ΔAIC_{diff} = -97.6), distance to the nearest road (0.150 ± 0.024, ΔAIC_{diff} = -36.1), bog (-0.490 ± 0.110, ΔAIC_{diff} = -17.7), old (-0.291 ± 0.088, ΔAIC_{diff} = -8.89), and mid-aged forest (0.233 ± 0.079, ΔAIC_{diff} = -6.65) (ESM3 Table 2, ESM3 Figure 1 and 2). Young forest (0.130 ± 0.089, ΔAIC_{diff} = -0.15), tree-rich bogs (-0.214 ± 0.155, ΔAIC_{diff} = -0.10), and NDVI (-0.028 ± 0.024, ΔAIC_{diff} = -0.55) did not affect litter survival (ESM3 Table S2, ESM3 Figure S1 and S2). Note that we reversed the sign of the estimates for the 'distance to' variables to facilitate interpretation; i.e. negative values imply avoidance, positive values indicate positive selection.

Conclusions – The results indicate that habitat selection on the landscape scale is also an important component of litter survival (ESM3 H1), and that successful mothers selected for areas close to human habitation whereas unsuccessful mothers avoided human habitation (ESM3 H2). In general, patterns in second and third-order resource selection appeared to be very similar, for both successful and unsuccessful mothers. One important question, however, remains unanswered; i.e. what is the underlying mechanisms for the selection for areas close to human habitation on the landscape scale? Two complimentary mechanism may explain this. Firstly,

successful mothers with a litter may actively shift their home range to areas of high human footprint, whereas unsuccessful mothers show higher site fidelity. Annual home range drift and low levels of site fidelity have been documented in several large mammals [3], including brown bears[4]. Such shifts are typically explained by variation in food availability [4]. However, other ecological conditions could explain such patterns as well (e.g. human disturbance) [5], including infanticide risk. Secondly, dispersal in brown bears is male biased and female brown bears form matrilineal assemblages [6]. Older, more experienced and dominant mothers may express more site fidelity than younger ones, and force younger and less dominant females to less favorable areas (e.g. close to human disturbance). Such mechanism would indicate a mismatch between recent rapid human population growth and encroachment in wildlife habitat and the evolution of reproductive strategies. Both mechanisms warrant further research on this topic.

ESM3 Table S1. Candidate models for second-order resource selection functions of female brown bears during the mating season in south-central Sweden (2005-2012), ranked according to the second-order bias corrected Akaike Information Criterion difference (ΔAIC_c) and weight (AIC_{cw}) values. '✓' indicates the inclusion of a landscape variable as a main term in a candidate model, '*' indicates the inclusion of the interaction term 'litter survival' with a certain landscape variable. NDVI = Normalized Difference Vegetation Index.

Model selection diagnostics			Landscape variables										
Rank	ΔAICc	AICcw	Distance to the nearest *forest road*	Distance to the nearest *road*	Distance to the nearest *habitation*	NDVI	Bog	Tree rich bog	Clearcut	Young forest	Mid-aged forest	Old forest
1	0.0	1	✓ *	✓ *	✓ *	✓ *	✓ *	✓ *	✓ *	✓ *	✓ *	✓ *
3	56.3	0	✓ *	✓ *	✓ *	✓ *	✓	✓	✓ *	✓	✓	✓ *
2	157.0	0	✓	✓ *	✓ *	✓ *	✓	✓	✓ *	✓ *	✓ *	✓ *
4	316.6	0	✓	✓	✓ *	✓	✓	✓	✓	✓	✓	✓ *
5	844.9	0	✓	✓	✓	✓	✓	✓	✓	✓	✓	✓
7	1595.7	0	✓	✓ *	✓ *							
6	1597.5	0	✓ *	✓ *	✓ *							
8	1621.7	0	✓	✓	✓ *							
9	1639.0	0				✓	✓ *	✓ *	✓ *	✓ *	✓ *	✓ *
10	1836.5	0				✓ *	✓ *	✓ *	✓ *	✓ *	✓ *	✓ *
11	1999.5	0				✓ *	✓	✓	✓ *	✓ *	✓ *	✓ *
12	2129.8	0				✓ *	✓	✓	✓ *	✓	✓	✓ *
13	2167.6	0				✓	✓	✓	✓	✓	✓	✓
14	2602.9	0	✓	✓	✓							
15	3534.0	0										

ESM3 Table S2. Most parsimonious candidate model to evaluate second-order resource selection of female brown bears in relation to litter fate (survival/mortality) during the mating season in south-central Sweden (2005-2012). NDVI = Normalized Difference Vegetation Index. '*' indicates statistically significant model terms (i.e., 0 not included in the 95% confidence interval). Note that we reversed the sign of parameter estimates of the 'distance to' variables to facilitate interpretation; positive values indicated 'selection for' whereas negative values indicated 'avoidance'.

**Model term**	**β**	**σ**	**95% confidence interval**		
			Lower level	***Upper level***	
Intercept	-0.356	0.075	-0.504	-0.208	*
Survival vs. Mortality	-0.041	0.080	-0.198	0.115	
Distance to the nearest forest road	-0.009	0.021	-0.032	0.050	
Distance to the nearest road	-0.375	0.020	0.336	0.414	*
Distance to the nearest habitation	-0.116	0.020	0.077	0.155	*
Bog (1 vs 0)	-0.652	0.086	-0.821	-0.483	*
Tree rich bog (1 vs 0)	0.338	0.124	0.095	0.582	*
Clearcut (1 vs 0)	-0.478	0.101	-0.675	-0.280	*
Young forest (1 vs 0)	0.453	0.072	0.311	0.595	*
Mid-aged forest (1 vs 0)	0.362	0.065	0.235	0.489	*
Old forest (1 vs 0)	0.638	0.072	0.497	0.779	*
NDVI	0.184	0.019	0.146	0.222	*
Distance to the nearest forest road * Survival	-0.308	0.025	0.258	0.358	*
Distance to the nearest road * Survival	0.150	0.024	-0.198	-0.102	*
Distance to the nearest habitation * Survival	0.540	0.025	-0.588	-0.492	*
Bog * Survival	-0.490	0.110	-0.706	-0.274	*
Tree rich bog * Survival	-0.214	0.155	-0.518	0.090	
Clearcut * Survival	1.144	0.116	0.917	1.372	*
Young forest * Survival	0.130	0.089	-0.044	0.304	
Mid-aged forest * Survival	0.233	0.079	0.078	0.388	*
Old forest * Survival	-0.291	0.088	-0.465	-0.118	*
NDVI * Survival	-0.028	0.024	-0.075	0.018	

ESM3 Figure S1. Parameter estimates and confidence intervals (β ± 1.96·se) of model variables included in the most parsimonious model to evaluate resource selection of female brown bears that experienced litter survival (black) and complete litter loss (grey) during the mating season in south-central Sweden (2005-2012). Positive values indicate selection, negative values indicate avoidance. We reversed the sign of the distance to the nearest 'road', 'forest road', and 'habitation' to facilitate interpretation. NDVI = Normalized Difference Vegetation Index, TRB = tree rich bog, Old = old forest, Mid aged = mid-aged forest, Young = young forest. We scaled all continuous variables around mean = 0 and variance = 1 to facilitate comparison.

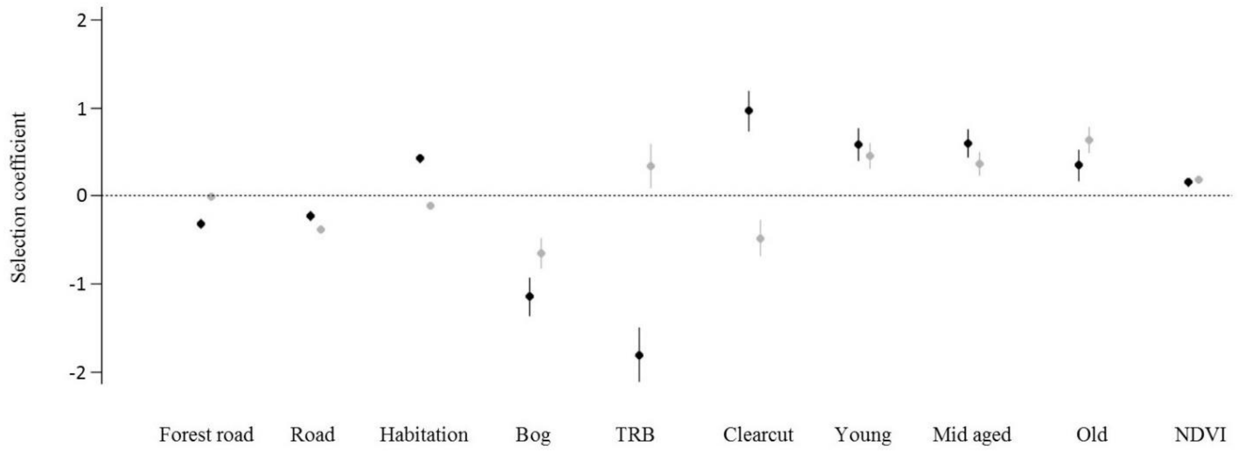

ESM3 Fig S2. Relative importance (ΔAIC_{diff}) of the interaction term ‘litter survival’ on the landscape variables in the most parsimonious model to assess second order resource selection of female brown bears that experience litter survival (N = 18) and complete loss (N = 11) during the mating season in south-central Sweden (2005-2012). $\Delta AIC_{diff} > 4$ (horizontal red line) supports the inclusion of the interaction term ‘litter survival’ on landscape variables. We reversed the sign of the ΔAIC_{diff} values to facilitate interpretation: high values indicate high importance. TRI = terrain ruggedness at the local scale, TRI1000 = terrain ruggedness at the landscape scale, Old = old forest.

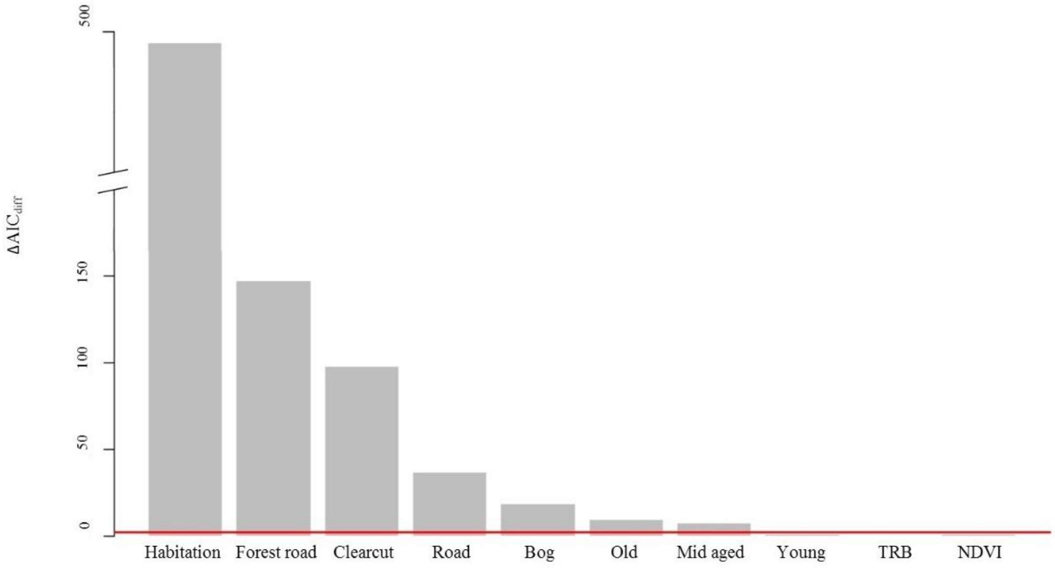

References

[1] Johnson, D.H. 1980 The comparison of usage and availability measurements for evaluating resource preference. *Ecology* **61**, 65-71.

[2] Manly, B., McDonald, L., Thomas, D.L., McDonald, T.L. & Erickson, W.P. 2002 *Resource selection by animals: statistical design and analysis for field studies*. Dordrecht, Kluwer Academic Publishers.

[3] Börger, L., Dalziel, B.D. & Fryxell, J.M. 2008 Are there general mechanisms of animal home range behaviour? A review and prospects for future research. *Ecology Letters* **11**, 637-650. (doi:10.1111/j.1461-0248.2008.01182.x).

[4] Edwards, M.A., Nagy, J.A. & Derocher, A.E. 2009 Low site fidelity and home range drift in a wide-ranging, large Arctic omnivore. *Animal Behaviour* **77**, 23-28. (doi:http://dx.doi.org/10.1016/j.anbehav.2008.09.025).

[5] Sorensen, A.A., Stenhouse, G.B., Bourbonnais, M.L. & Nelson, T.A. 2015 Effects of habitat quality and anthropogenic disturbance on grizzly bear (Ursus arctos horribilis) home-range fidelity. *Canadian Journal of Zoology* **93**, 857-865. (doi:10.1139/cjz-2015-0095).

[6] Zedrosser, A., Støen, O.-G., Sæbø, S. & Swenson, J.E. 2007 Should I stay or should I go? Natal dispersal in the brown bear. *Animal Behaviour* **74**, 369-376. (doi:http://dx.doi.org/10.1016/j.anbehav.2006.09.015).
