# Supplementary material for: Human shields mediate sexual conflict in a top predator
Source: Proc Biol Sci. 2016 Jun 29;283(1833):20160906. doi: 10.1098/rspb.2016.0906 (PMC4936045; doi:10.1098/rspb.2016.0906)
Supplement: Electronic Supplementary Material 4 - Habitat use [file rspb20160906supp4.pdf]

Electronic Supplementary Material 4 – Habitat use

GPS relocation data can be used to estimate habitat use or the 'use distribution', i.e., the distribution of animal relocations on the landscape covariate space, irrespective of the covariate availability [1, 2]. Habitat use can provide a good measure of animal exposure to specific resources, irrespective of resource availability. Measures of habitat use thus provide complimentary information to the measures of habitat selection we present in the main document. Therefore, we reformulated our main hypotheses to ESM4 H1) habitat use is an important component of offspring survival, and ESM4 H2) successful mothers (i.e., litter survival) generally use areas closer to human footprint than unsuccessful mothers (i.e., litter mortality).

Methods – We contrasted the GPS relocation datasets of successful mothers (1) with unsuccessful mothers (0) in a logistic mixed effect regression model, with 'bear ID' as a random factor. The model results will thus provide information about the probability that any given GPS relocation originates from a successful (1) or unsuccessful (0) mother. The model only included influential variables ($\Delta AIC_{diff} > 4$) of the full model as presented in the main article, and 'bear identity' as a random factor on the intercept. We did not include 'year' as random factor because of its' relatively low influence and to improve model convergence. We compared the full model with a null model only. There was no reason to include 'litter survival' as an interaction term, because litter survival was inherently included in the response variable.

Results – The full model ($AIC_{cw} = 1$) outcompeted the null model ($AIC_{cw} = 0$, $\Delta AIC_c = 791.7$). Habitat use differed between successful and unsuccessful mothers with respect to distance to the nearest road (-1.237 ± 0.110) and habitation (-0.856 ± 0.052), clearcut (1.257 ± 0.176), old forest

(0.517 ± 0.09) (ESM4 Table 1). The use of bogs (-0.052 ± 0.156) was similar between successful and unsuccessful mothers (ESM4 Table 1).

Conclusions –Habitat use is indeed an important component of litter survival in female brown bears (ESM4 H1), and successful mothers use areas closer to human footprint than unsuccessful mothers (ESM4 H2). Irrespective of habitat availability, successful mothers used areas further closer to human habitation and roads. Successful mothers used old forest and clearcuts more often than unsuccessful mothers. These results support that our main findings are valid, irrespective of variations in human habitation habitat availability within home-ranges.

ESM4 Table S1. Most parsimonious candidate model to evaluate habitat use of female brown bears that experienced cub survival (1) and cub loss (0) during the mating season in south-central Sweden (2005-2012).

**Model term**	**β**	**σ**	**95% confidence interval**		
			Lower level	***Upper level***	
Intercept	17.181	2.380	12.516	21.846	*
Distance to the nearest road	-1.237	0.110	-1.453	-1.021	*
Distance to the nearest habitation	-0.856	0.052	-0.957	-0.755	*
Bog	-0.052	0.156	-0.358	0.254	
Clearcut	1.257	0.176	0.912	1.601	*
Old forest	0.517	0.090	0.340	0.693	*

References

[1] Lele, S.R., Merrill, E.H., Keim, J. & Boyce, M.S. 2013 Selection, use, choice and occupancy: clarifying concepts in resource selection studies. *Journal of Animal Ecology* **82**, 1183-1191. (doi:10.1111/1365-2656.12141).

[2] Johnson, D.H. 1980 The comparison of usage and availability measurements for evaluating resource preference. *Ecology* **61**, 65-71.
